# Beyond Bouma's window: How to explain global aspects of crowding?

**DOI:** 10.1371/journal.pcbi.1006580

**Published:** 2019-05-10

**Authors:** Adrien Doerig, Alban Bornet, Ruth Rosenholtz, Gregory Francis, Aaron M. Clarke, Michael H. Herzog

**Affiliations:** 1 Laboratory of Psychophysics, Brain Mind Institute, École Polytechnique Fédérale de Lausanne (EPFL), Lausanne, Switzerland; 2 Department of Brain and Cognitive Sciences, Computer Science and Artificial Intelligence Laboratory, MIT, Cambridge, MA, United States of America; 3 Department of Psychological Sciences, Purdue University, West Lafayette, IN, United States of America; 4 Laboratory of Computational Vision, Psychology Department, Bilkent University, Ankara, Turkey; Technische Universitat Chemnitz, GERMANY

## Abstract

In crowding, perception of an object deteriorates in the presence of nearby elements. Although crowding is a ubiquitous phenomenon, since elements are rarely seen in isolation, to date there exists no consensus on how to model it. Previous experiments showed that the global configuration of the entire stimulus must be taken into account. These findings rule out simple pooling or substitution models and favor models sensitive to global spatial aspects. In order to investigate how to incorporate global aspects into models, we tested a large number of models with a database of forty stimuli tailored for the global aspects of crowding. Our results show that incorporating grouping like components strongly improves model performance.

## Author summary

Visual crowding highlights interactions between elements in the visual field. For example, an object is more difficult to recognize if it is presented in clutter. Crowding is one of the most fundamental aspects of vision, playing crucial roles in object recognition, reading and visual perception in general, and is therefore an essential tool to understand how the visual system encodes information based on its retinal input. Hence, classic models of crowding have focused only on local interactions between neighboring visual elements. However, abundant experimental evidence argues against local processing, suggesting that the global configuration of visual elements strongly modulates crowding. Here, we tested all available models of crowding that are able to capture global processing across the entire visual field. We tested 12 models including the Texture Tiling Model, a Deep Convolutional Neural Network and the LAMINART neural network with large scale computer simulations. We found that models incorporating a grouping component are best suited to explain the data. Our results suggest that in order to understand vision in general, mid-level, contextual processing is inevitable.

## Introduction

**Fig 1:**
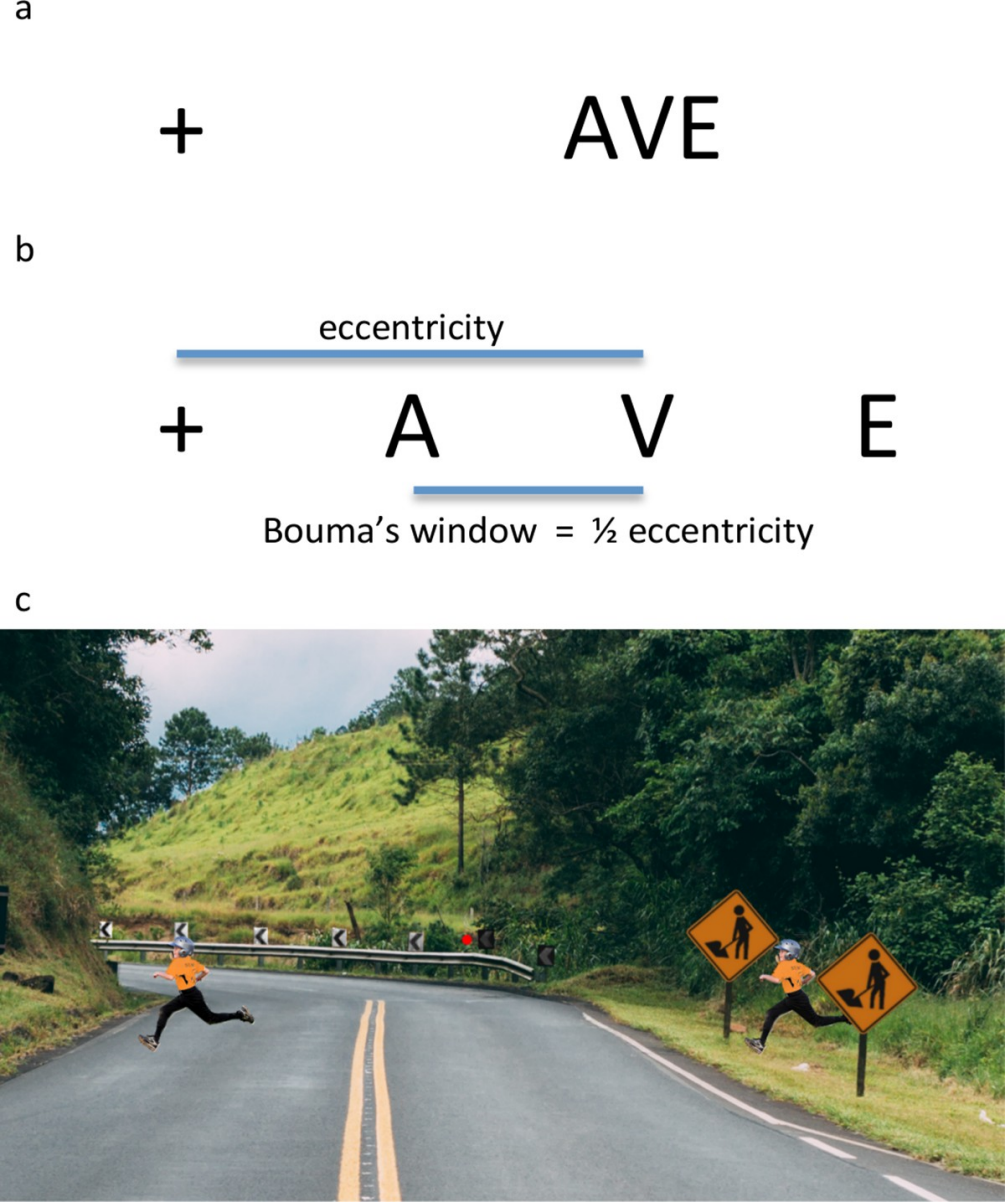
Crowding. **a.** In crowding, the perception of a target element deteriorates in the presence of nearby elements. When fixating the left cross, the target letter V on the right is hard to identify because of the nearby flankers. **b.** The task is easier than in (a), because the flankers are further away from the target letter V. Bouma’s law states that crowding occurs only when flankers are sufficiently close to the target, within the so-called Bouma’s window. **c.** Crowding is a ubiquitous phenomenon since elements are rarely seen in isolation. For example, when fixating the central red dot, the child on the left is easier to detect because it is not surrounded by nearby flankers, as is the child on the right.

When an element is presented in the presence of nearby elements or clutter, it becomes harder to perceive, a well-known effect called crowding. One of the main characteristics of crowding is that the element itself is not invisible, contrary to contrast- and backward-masking; rather its features appear jumbled and distorted (Fig 1). Crowding is a ubiquitous phenomenon because elements are rarely encountered in isolation in everyday situations (Fig 1C). Thus, understanding crowding is crucial for understanding vision in general.

For about half a century, the consensus was that flankers interfere with a target element only when placed within a spatially restricted window around the target, the so-called Bouma law (Fig 1B; [1–4]):

SizeofBouma’swindow≈0.5*eccentricity

**Fig 2:**
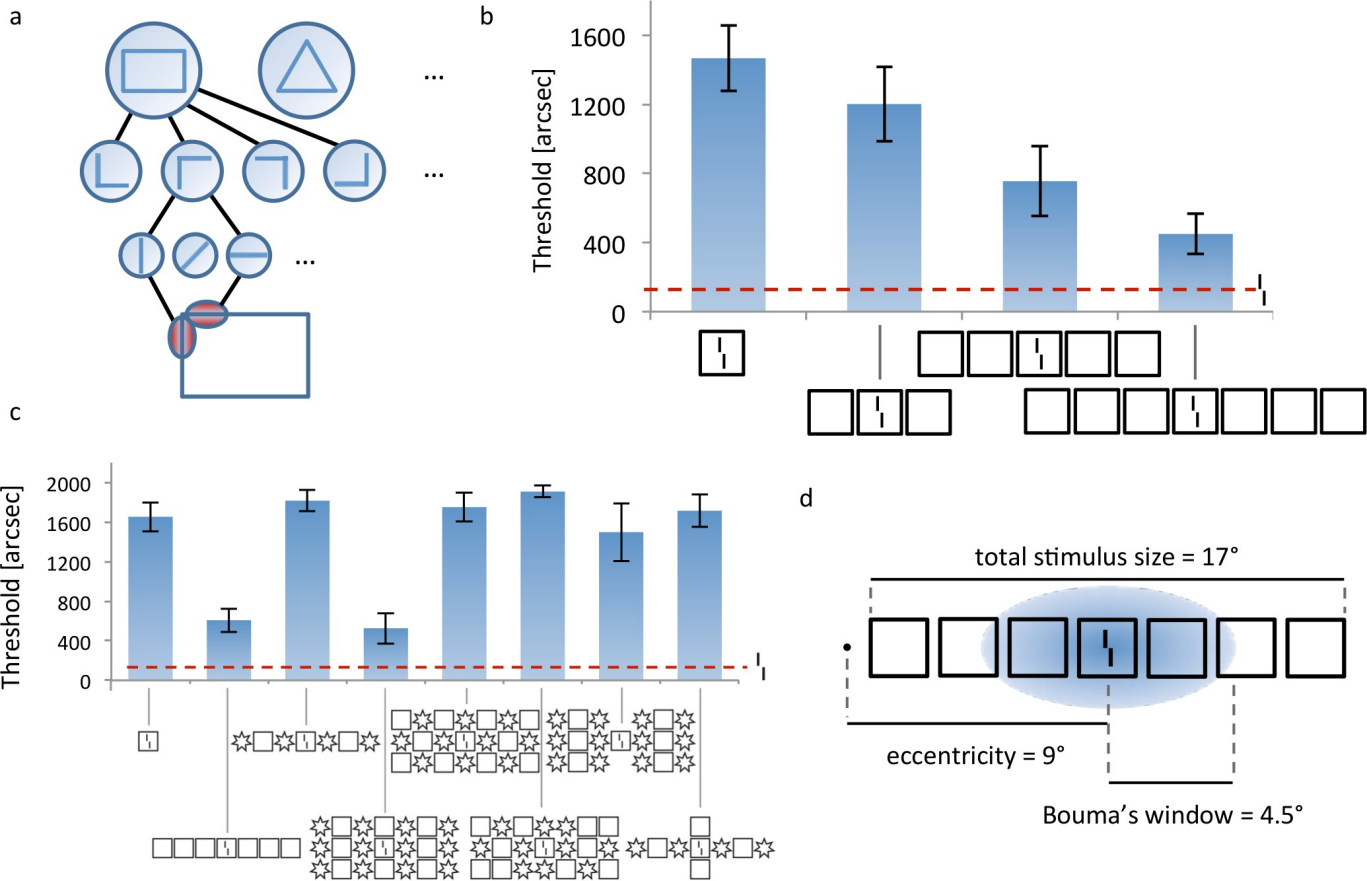
**a. Standard view of visual processing.** First, edges are detected by low-level neurons with small receptive fields. Higher level neurons pool signals from lower level neurons in a hierarchical, feedforward manner, creating higher level representations of objects by combining low-level features [25,26]. For example, two low-level edge detectors may be combined to create a “corner” representation. Four such corner detectors can be assembled to create a rectangle representation. Receptive field size naturally increases along this pathway since, for example, a rectangle covers larger parts of the visual field than the lines making up the rectangle. **b. Uncrowding.** Observers performed a vernier discrimination task. The y-axis shows the threshold for which observers correctly discriminate the vernier offset in 75% of trials (so performance is good when the threshold is low). First, only a vernier is presented, an easy task (performance for this condition is shown as the dashed horizontal line). Then, a flanking square is added making the task much more difficult (leftmost stimulus). This is a classic crowding effect. Importantly, adding more flanking squares improved performance gradually, i.e., performance improved the more squares are presented [19]. We call this effect uncrowding. **c. The global configuration** of the entire stimulus determines crowding. Performance is strongly affected by elements far away from the target as shown in these examples [15]. **d. Performance is not determined by local interactions only**. In this display, fine-grained vernier acuity of about 200” depends on elements as far away as 8.5 degrees—a difference of two orders of magnitude, extending far beyond Bouma’s window.

Classic models of crowding proposed that early visual areas, such as V1, process the features of stimuli with high precision. Crowding occurs when neural signals are pooled along the visual hierarchy, e.g., when V2 neurons pool neural signals from V1 neurons [5]. Hence, in line with classic hierarchical feedforward processing (Fig 2A), crowding may be seen as a natural consequence of object recognition in the visual system. For example, a hypothetical neuron coding for a square might respond to signals from neurons coding for the lines making up the square. In order to achieve translational invariance, the square neuron is sensitive to lines all over its receptive field and pools this information in order to decide whether a square is present. According to this logic, crowding occurs when elements that do not belong to the same object are pooled. In this sense, crowding is an unwanted by-product of object recognition and, for this reason, a bottleneck of vision (for reviews, see [2,6]). Other models have proposed that performance in crowding deteriorates because features of the target are substituted for features of the flanking elements [4,7]. As mentioned, all these models are local in the sense that crowding is determined by nearby elements only. Based on these two lines of thought, pooling and substitution, researchers have suggested that with more flankers performance deteriorates because more irrelevant features are pooled or substituted.

The understanding of crowding has largely changed in the last decade. For example, it has been shown that detailed information can survive crowding [8,9]. Crowding occurs in the fovea and is not restricted to the periphery, contrary to earlier proposals [10,11]. Most importantly for the present discussion, performance depends on elements far beyond Bouma’s window. For example, in supercrowding, elements outside of Bouma’s window decrease performance beyond the decrement arising from elements within the window [12]. Surprisingly, adding flankers can even reduce crowding, and such uncrowding effects can depend on elements outside of Bouma’s window (Fig 2; [10,13–17], review: [18]). For example, observers performed a vernier discrimination task. When a surrounding square was added to the vernier, the task became much more difficult: a classic crowding effect. However, adding more flanking squares improved performance gradually, i.e., performance improved the more squares were presented ([19]; Fig 2B). The entire line of squares extends over 17 degrees in the right visual field, while the single vernier offset threshold is less than 200” (Fig 2D). Hence, performance is not exclusively determined by local interactions: fine-grained vernier acuity in the range of about 200” depends on elements as far away as 8.5 degrees—a ratio of two orders of magnitude, extending far beyond Bouma’s window. Moreover, performance depends on the overall configuration [20]. For example, in three-by-seven displays of squares and stars (Fig 2C), a shift of the central row changes performance strongly (Fig 2C, 4^th^ and 5^th^ configurations). Similar effects were found with stimuli other than verniers [21,22], as well as in auditory [23] and haptic crowding [24].

**Fig 3:**
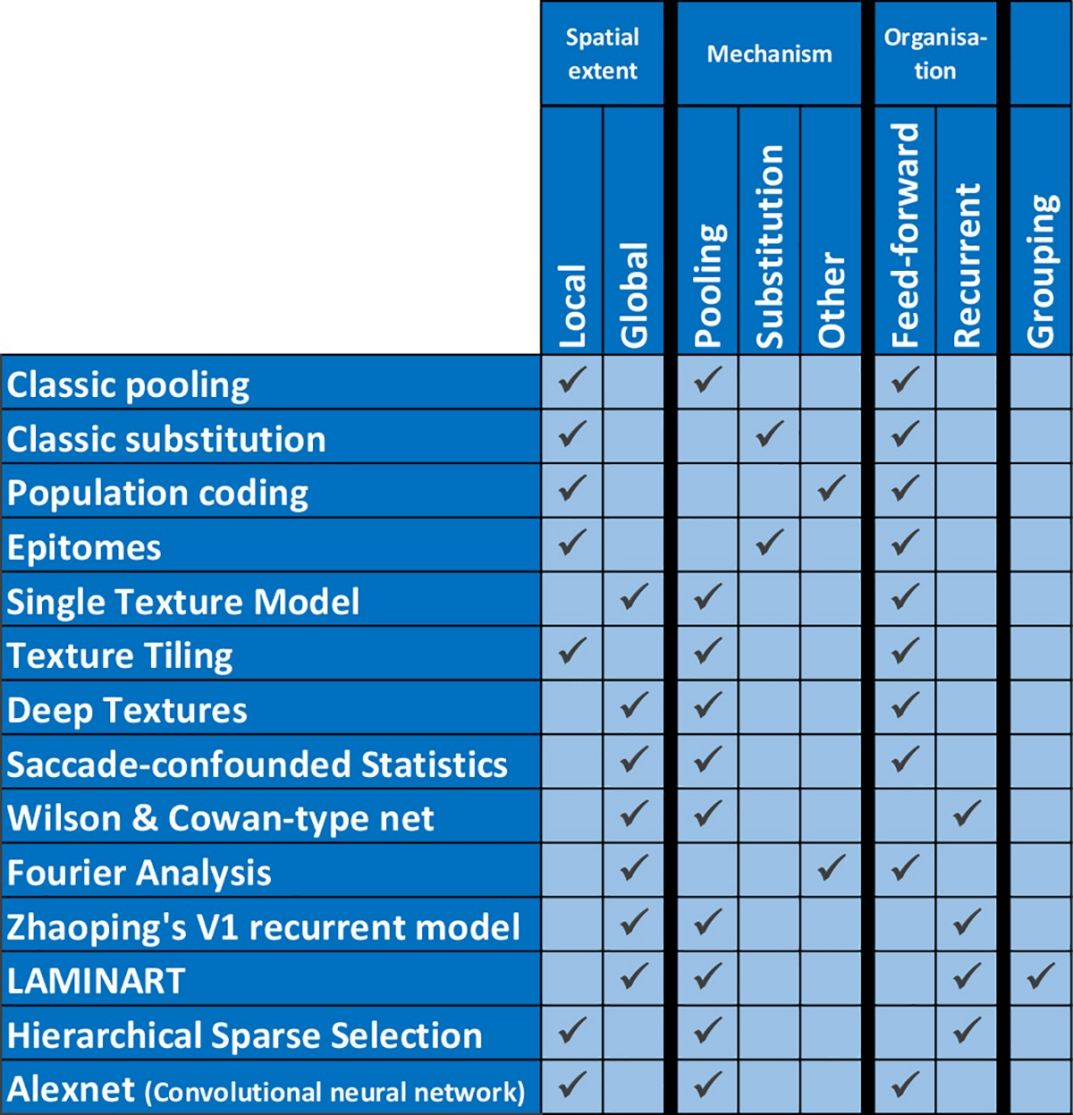
Tested models and their characteristics. Models may integrate information locally or globally, and the interference mechanism may be pooling, substitution, or other. Models are feed-forward or recurrent, and may or may not compute grouping-like aspects of the stimulus. The aim of the current work is to investigate which models can explain the global effects of crowding.

Because they cannot produce long-range effects, local models cannot explain the global aspects of crowding. Here, we tested which global models, integrating information across large parts of the visual field, can explain global effects on crowding (see Fig 3 for a list). We also tested the most prominent local models to verify our hypothesis that local models are inadequate to explain global aspects of crowding.

The models that we tested differ with respect to four criteria:

Spatial extent: Local vs. Global. In a local model, elements far from the target do not exert any effects on the target. By contrast, in a global model, any element in the visual field may potentially interfere with target processing.

### Mechanism of interference: Pooling, substitution, or other?

Organisation: Feed-forward (features at a given level are only affected by lower level features) vs. recurrent processing (features at a given level can be affected by lower or higher level features).

Grouping component: Does the model incorporate a grouping component? Certain models explicitly compute grouping-like aspects by determining which low-level elements should belong to the same higher level group. Only elements within a group interfere with each other.

## Methods

**Fig 4:**
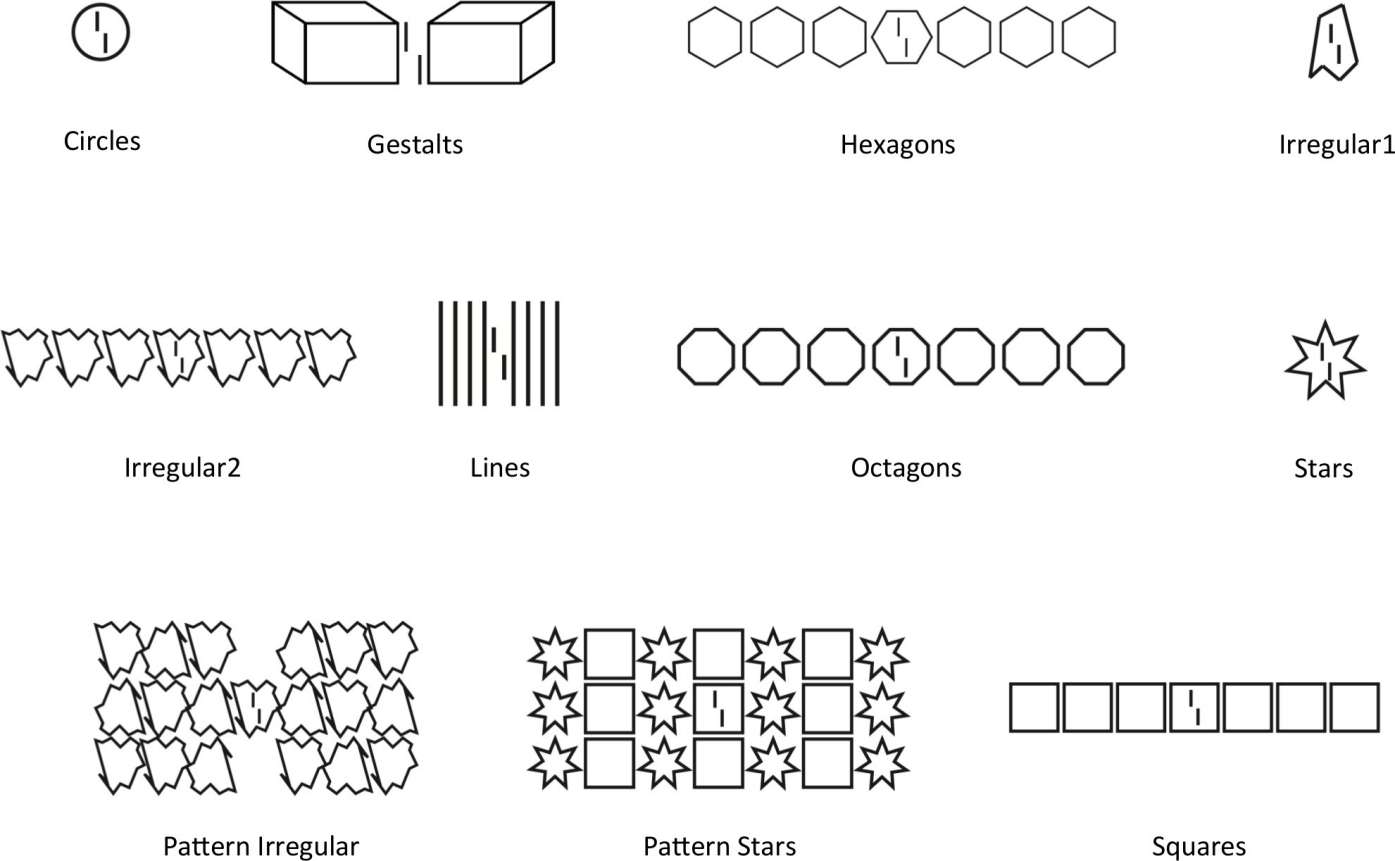
Stimulus categories. We used 40 different stimuli from 11 different categories. The task was always to report the offset direction of the central vernier. This figure shows one example from each category. The stimulus database is tailored to test for global effects such as uncrowding. Human data was taken from previous work [10,11,15,17,19,20]. Human and model results are summarized in the discussion (Fig 14 shows the results for all stimuli and models).

To test the models, we used human data from previous work exploring the crowding/uncrowding phenomena [10,11,15,17,19,20]. The stimulus database comprises 40 different stimuli belonging to 11 different categories: circles, Gestalts, hexagons, irregular1, irregular2, lines, octagons, patternIrregular, patternStars, squares and stars. An example of each category is shown in Fig 4. Behavioral results can be found in the original papers (listed in Fig 14). In each category, we have the vernier target alone, plus crowding and uncrowding configurations. All the stimuli are shown in Fig 14 and behavioural results can be found in the original papers. With a few exceptions (see details in the results section), we ran each model on all stimuli. For some models, we could not use the entire database because computation time was too long (deep convolutional networks, LAMINART, Texture Tiling Model), or because the model was not adapted to accommodate certain kinds of stimuli (Population Coding). Human and model results are summarized in the discussion (Figs 14 & 15). All the code we used is available online at https://github.com/adriendoerig/beyond-boumas-window-code. All the results can be found at https://github.com/adriendoerig/beyond-boumas-window-results.

There are two fundamentally different approaches to measure model performance. First, a linking hypothesis may be used to relate model output to performance (both are scalar numbers). For example, template matching computes how similar the model output is to the target image. If they are similar, performance is good. The second, textural approach is used to quantify performance in textural models. The idea is that peripheral vision is ambiguous because information is compressed by summary statistics. If a model uses a proper algorithm for representing these ambiguities, presenting the processed image in the fovea should lead to similar human performance as presenting the original unprocessed image in the periphery [27]. Accordingly, to measure the performance of textural algorithms, the stimuli are fed through a texture synthesis procedure. Then, observers freely examine the output image and report vernier orientation. If this task is easy, performance is good. For each model, we used the linking hypothesis proposed by the original authors when available. When this was not possible (for example for Alexnet, which has never been applied to crowding results before), we detail which linking hypothesis we used in the corresponding section. In the following, we present, first, textural models and, second, models using a linking hypothesis.

An important point is that different readouts lead to different results. Hence, the different methods of model evaluation used here could affect our results. However, we are mainly interested in qualitative rather than quantitative comparisons and the readout functions we used cannot confuse crowding and uncrowding. More specifically, the readout processes we use produce results monotonically linked to the model outputs. Hence, they cannot confuse uncrowding cases (a U-shape function where the vernier alone condition leads to good performance, a single flanker deteriorates performance, and multiple flankers lead again to good performance) with cases that do not show uncrowding (a monotonic function where the vernier alone condition leads to good performance, a single flanker deteriorates performance, and multiple flankers deteriorate performance even more).

Because different models were evaluated differently, it was impossible to come up with one performance measure and to compare models via something like the Akaike Information Criterion. However, despite this variety of performance measures, our results are qualitatively unambiguous: each model either is capable of producing uncrowding, or it is not. We took the parameters directly from the original models whenever possible. Otherwise, we tried our best to search the parameter space (see results). We cannot exclude that other combinations of parameters fit the dataset better. However, we will argue that the models that cannot produce uncrowding fail to do so for principled reasons, and not because of poor parameter choices (see discussion).

## Results

### Texture-like models

The following models are based on texture analysis. The outputs are images, and the texture method is applied as described in the methods.

#### Epitomes

In the Epitomes model, described by Jojic et al. [28], large repeating patterns are summarized by small repeated representative image patches. Repeated patterns are substituted with their exemplars. The original image can subsequently be retrieved with good accuracy from the compressed representation, even though neighboring features encoded in the same patch are mingled. Epitomes are effectively a “substitution” model that exploits regularities. Although this model was not proposed as a model of crowding, it embodies many of the key characteristics of local pooling and substitution models.

**Fig 5:**
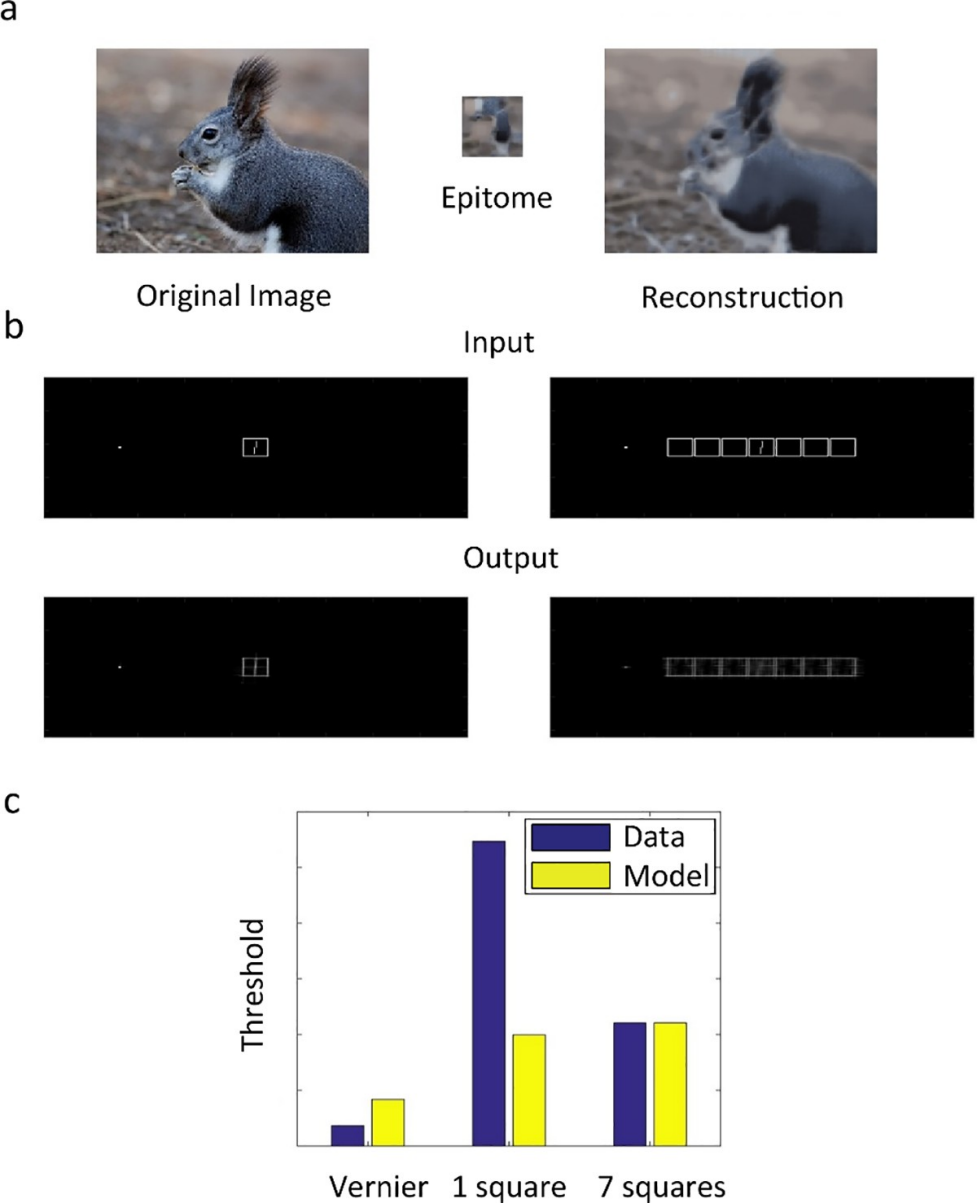
Epitomes. **a.** Illustration of the epitome model. An image (left) is compressed into an epitome (center), a summary of local features. The image on the right is reconstructed from the epitome. **b.** As an example for the classic texture evaluation, we show the stimulus and reconstructed image for the 1- and 7-square conditions. Human vernier offset thresholds are better for the 1-square than the 7-square condition. The model does not produce uncrowding because vernier offset direction in the output is not easier to make out in the 7-square than in the 1-square case (according to the authors’ judgment). **c.** Example for our performance measure. Human and model thresholds (see main text for how model threshold was computed) for vernier alone, single square and 7 squares conditions. The 7-square threshold is higher than the 1- square threshold, in contrast with human performance. Note: the model outputs a number quantifying how different the left and right vernier offset versions of the input are (so the higher this difference, the better the performance). To make comparison with the human threshold easier, we applied the following monotonic transformation to the output: “threshold-like output” = 1/“raw output”. Then, we scaled the result to be in the same range as the human results. This monotonic re-scaling cannot change the conclusions because monotonic outputs are mapped on monotonic performance and the same is true for U-shaped functions (see methods).

Using the author’s code available online (http://www.vincentcheung.ca/research/sourcecode.html) we ran the model on all stimuli with the original parameters (designed to optimize image reconstruction accuracy for natural images and texture overlays). To evaluate performance, we used the texture evaluation method with the authors as subjects, analysing the results qualitatively (see methods). In addition, we computed the model threshold as

$$\int \int_{x,y}|leftStim(x,y))|-|\mathrm{rightStim}(x,y))|dxdy$$

where leftStim(x,y) is the normalized intensity of pixel (x,y) in the left vernier offset version of the output. Effectively, this equation quantifies how different the normalized output images are for the left and the right vernier offset versions of the stimulus. If they are very different, the task is easy. Consistently across the dataset, the model successfully produces crowding but not uncrowding: performance was always worse when adding more flankers (Fig 5). We suggest that the model cannot explain uncrowding because it compresses information from local regions of the image, ignoring global structure.

#### Single texture model

**Fig 6:**
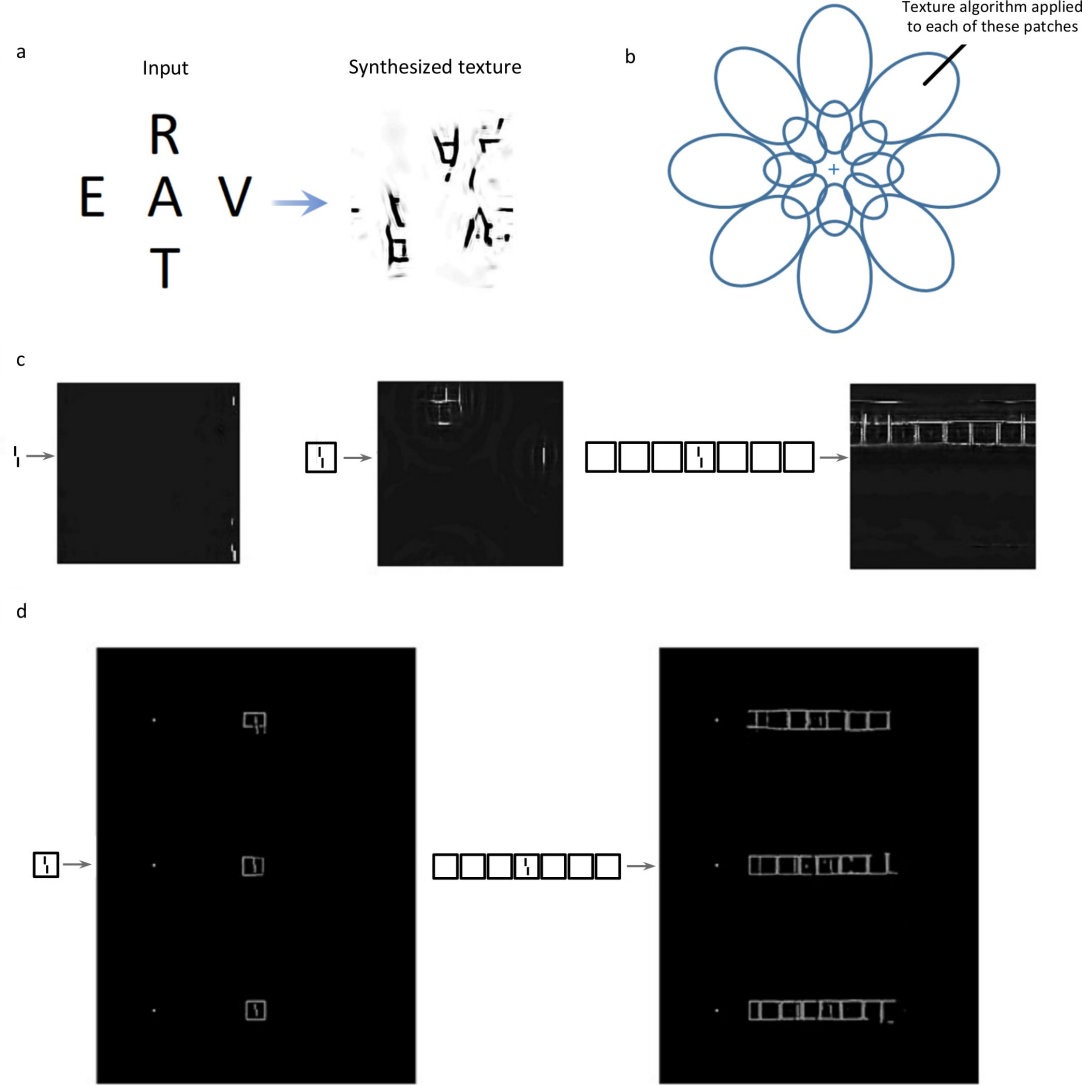
Texture Synthesis and Texture Tiling Model. **a.** A texture (right) synthesized from the input on the left using the Portilla & Simoncelli [29] summary statistics. The output resembles crowding. Pooling- and substitution-like effects occur. **b.** In the TTM, instead of applying the summary statistics process to the whole image at once, only local patches of the image are processed, yielding a local summary statistics model. The local patches are thought to reflect V2 receptive fields. **c.** Whole-field summary statistics. From left to right: stimuli and Portilla & Simoncelli textures for the vernier, 1-square and 7-square conditions. The vernier offset is easy to determine from the texture in the vernier alone condition, and slightly harder in the crowded condition (a right-offset is discernable in the middle top of the display). Across all data, the model consistently produces crowding, but no uncrowding, as exemplified in the right condition in which no offset is present at all. **d.** Texture Tiling model. The left column shows three synthesized examples from the 1-square condition. On the right is the 7-flanking squares case. The model cannot produce uncrowding: since the stimulus on the right is less crowded than the stimulus on the left in the human data, the direction of the vernier should be easier to make out on the right than on the left. However, this is not the case.

Portilla & Simoncelli [29] proposed a set of statistics capable of capturing key aspects of texture appearance to human vision (Fig 6A). Balas et al., [27] suggested an explanation of crowding in which peripheral vision might measure these texture statistics in pooling regions that overlap and tile the visual field. The intuition is that summary statistics provide an efficient way of extracting relevant information at low computational cost from natural images. Though Balas et al. proposed a model covering the entire visual field as described in the next subsection, they initially tested the predictions of a single pooling region, since texture synthesis procedures did not exist for multiple overlapping pooling regions. Each of their stimuli fell within a single Bouma-sized patch. They have since suggested that this shortcut of using a single pooling region, which greatly reduces computation time, can often suffice for texture-like stimuli that fall within a single pooling region [30].

Although the model was intended by Balas et al. to be applied only over a Bouma’s window-sized patch, here we applied it to the entire stimulus to see if this kind of texture synthesis could capture long-range interactions between the vernier and other elements. The texture statistics are computed from pixel intensities taken from the entire image. Using the code provided online by Portilla & Simoncelli (https://github.com/LabForComputationalVision/textureSynth), we created textures from all of our stimuli and the authors analyzed the results qualitatively using the texture measure (see Fig 6C for two examples). The model produces strong crowding: vernier offsets are harder to discriminate from the textures when flankers are present. However, the model cannot explain uncrowding: consistently across our whole dataset, uncrowded conditions are worse than crowded conditions for this model (Fig 6C). More elements always deteriorate performance. In their original contribution, Balas et al. seeded the texture synthesis algorithm using a low-pass, noisy version of the stimulus to reduce position noise. We also ran our stimuli using this method (see results repository online). While the output images became less distorted than without using the seed, it did not change the conclusion, because the target vernier remained much harder to detect in the textures synthesized from the uncrowded 7 flankers stimuli than from the crowded single flanker stimuli–i.e., there was no uncrowding.

#### Texture tiling model (TTM)

The TTM model was first described by Balas et al. [27], with its first full instantiation developed by Freeman & Simoncelli [31]. It computes summary statistics for overlapping local patches of the visual field, mimicking the way V2 receptive fields grow in size with eccentricity (Fig 6B). Balas, Rosenholtz and others have studied this model extensively, calling it the Texture Tiling Model (TTM; [32,33]). In a series of papers, this model explained well the local aspects of visual tasks such as crowding and visual search. We ran a selection of stimuli through the TTM model (circles, squares, and irregular1; code for the model accompanies [34]). Similarly to the previous textures, the results were analysed by the authors using the texture measure. Crowding was well captured, but uncrowding could not be explained by TTM (Fig 6D). The vernier was not better represented as the number of flankers increased.

We suggest that TTM alone cannot explain uncrowding because it is a sophisticated local mechanism that scrambles together neighboring elements. There is no mechanism allowing elements that do not share a pooling region with the target to directly affect the target representation. Our results suggest that neither pooling summary statistics over the entire stimulus nor pooling over previously tested local regions explain the behavioural results. If the whole field is used, uncrowding cannot occur because more elements mean more interference and thus worse performance. On the other hand, using local regions does not help because far away elements cannot improve performance in cases where humans show uncrowding.

#### Deep textures

**Fig 7:**
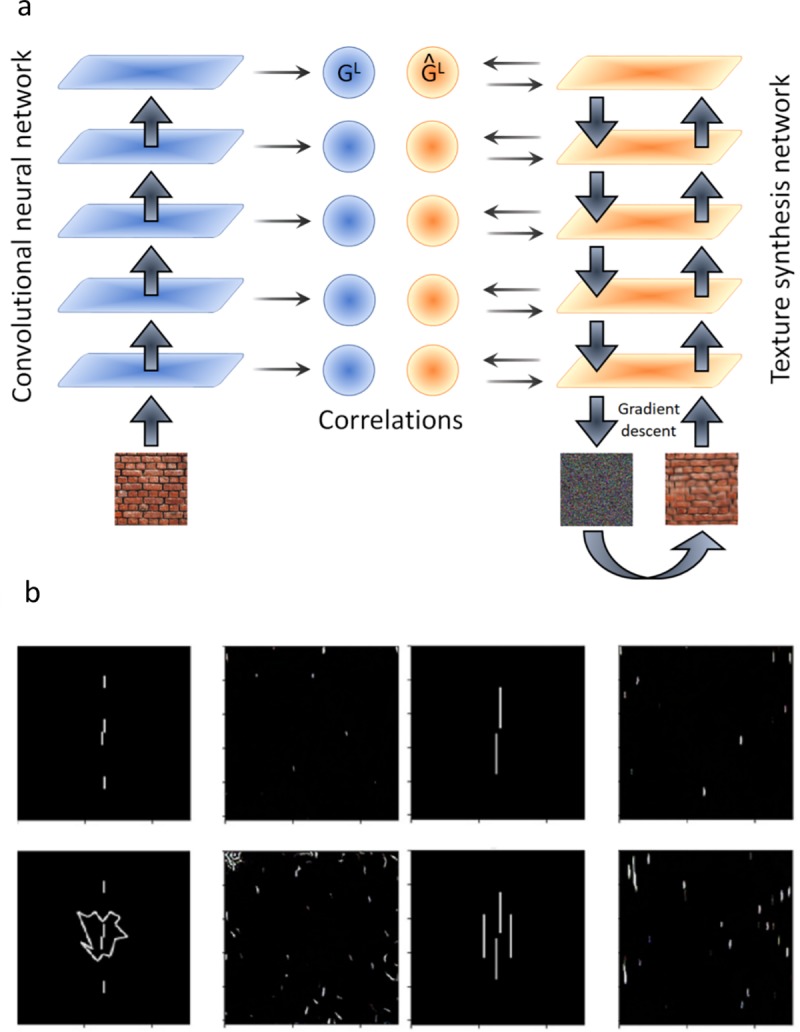
Deep textures. **a.** In the deep textures algorithm, the correlation between a deep neural network’s unit activities is used as a summary statistic. Textures are then synthesized to match that statistic. **b.** Original stimuli and textures synthesized from these stimuli using the deep textures algorithm by Gatys et al. [35]. The vernier offset is poorly visible, therefore, despite its clear success at synthesizing textures, the model in its present form in not suitable to model crowding with our stimuli. We tried different zooms on our stimuli but the results did not change.

Gatys and colleagues [35] used deep neural networks to create textures. The algorithm starts with a noise image and iteratively modifies it to match the correlations between neuron activities in a set of layers (Fig 7A). This procedure synthesizes textures that are often indistinguishable from the original image, creating true metamers [36]. Deep textures were not intended to be applied to images like our stimuli, nevertheless we were interested in seeing if they could handle them because one could think of deep textures as synthesizing textures based on learned features rather than on the hand-coded features of Portilla & Simoncelli [29]. Perhaps the learned features provide a better representation and thus do a better job of predicting crowding.

Using Gatys et al.’s code with their suggested set of parameters (https://github.com/leongatys/DeepTextures), we created textures of each stimulus in our database (Fig 7B shows a selection of examples). We first evaluated model performance by the texture measure performed by the authors. Since the results were much less clear than for the previous texture approaches, we also conducted a psychophysical experiment with naive participants. Five subjects performed the classic texture measure: they were first explained the texture synthesizing process and then were shown textures synthesized from our stimuli. They were asked to report if they thought the texture was synthesized from a left- or right-vernier stimulus. We used three categories of stimuli (Gestalts, squares and circles), with ten textures per stimulus (a total of 100 textures). Performance was at chance for all stimuli. Textures for the untested stimulus categories strongly resemble the tested categories (the vernier offset orientation is not visible in the textures, even for the vernier-alone condition). We tried different stimulus sizes, but this did not improve the results. In conclusion, despite its clear success at texture synthesis for natural images, the model in its present form is not suitable to study crowding with our stimuli.

Wallis et al. [37] have proposed a foveated model in which these deep statistics are computed over local image patches, just as the TTM computes Portilla and Simoncelli’s statistics over local patches. The code is not yet publicly available, so we did not test it explicitly, however, we believe it will not explain uncrowding for exactly the same reasons that the TTM does not handle uncrowding better than Portilla and Simoncelli’s whole field statistics: distant elements that are not in pooling regions around the target cannot affect the target representation.

### Models using a linking hypothesis

The following models all use a linking hypothesis to relate their output (a number) to human performance. Whenever possible, we used the same linking hypothesis as in the original contribution. When no linking hypothesis was available, we specify the method used.

#### Wilson & Cowan network with end-stopped receptive fields

Wilson & Cowan [38] proposed a mathematical model of simple cortical (excitatory and inhibitory) neurons interacting through recurrent lateral connexions. Variations of this kind of model have successfully accounted for visual masking data using stimuli similar to our lines category [39]. We used a similar neural network for our crowding stimuli. The model first convolves the input image with an on-center, off-surround receptive field mimicking processing by the LGN. Next, the input activations are fed into both an excitatory and an inhibitory layer of neurons, which are reciprocally connected such that the excitatory units excite the inhibitory units and the inhibitory units inhibit the excitatory units. Details of the model, its filters, and its parameters can be found in [39] and [40]. Although the filters are local, the strength of activity at any given pixel location partly depends on the global pattern of activity across the network because of the feedback connections. More generally, the feedback in the network functions like a discontinuity detector by enhancing discontinuities and suppressing regularities. Clarke, Herzog & Francis [41] applied this model to crowding stimuli, but it performed poorly and produced no uncrowding. For example, there was no difference between the stimuli in the Gestalts category and the length of the bars in the lines category had no effect at all on performance. Here, to improve the model, we replaced the classic receptive fields by end-stopped receptive fields so that each neuron is optimally activated only by stimuli of a specific length. There were three different sizes for the end-stopped receptive-fields, corresponding to the size of a vernier bar, the size of the whole vernier, and the size of the flankers. To measure performance for each stimulus, for each end-stopped receptive field size, we took as output the state of the excitatory layer after stabilization (40 time-steps) and cross-correlated it with the vernier alone output. The cross-correlations for each end-stopped receptive field size were summed to yield a single output number per stimulus. We then fitted a psychometric function on one class of stimuli (training set) and used this function to provide model performance for all other classes of stimuli (testing set). Apart from the end-stopped receptive fields modification, we used the same parameters as in Hermens et al. [39].

**Fig 8:**
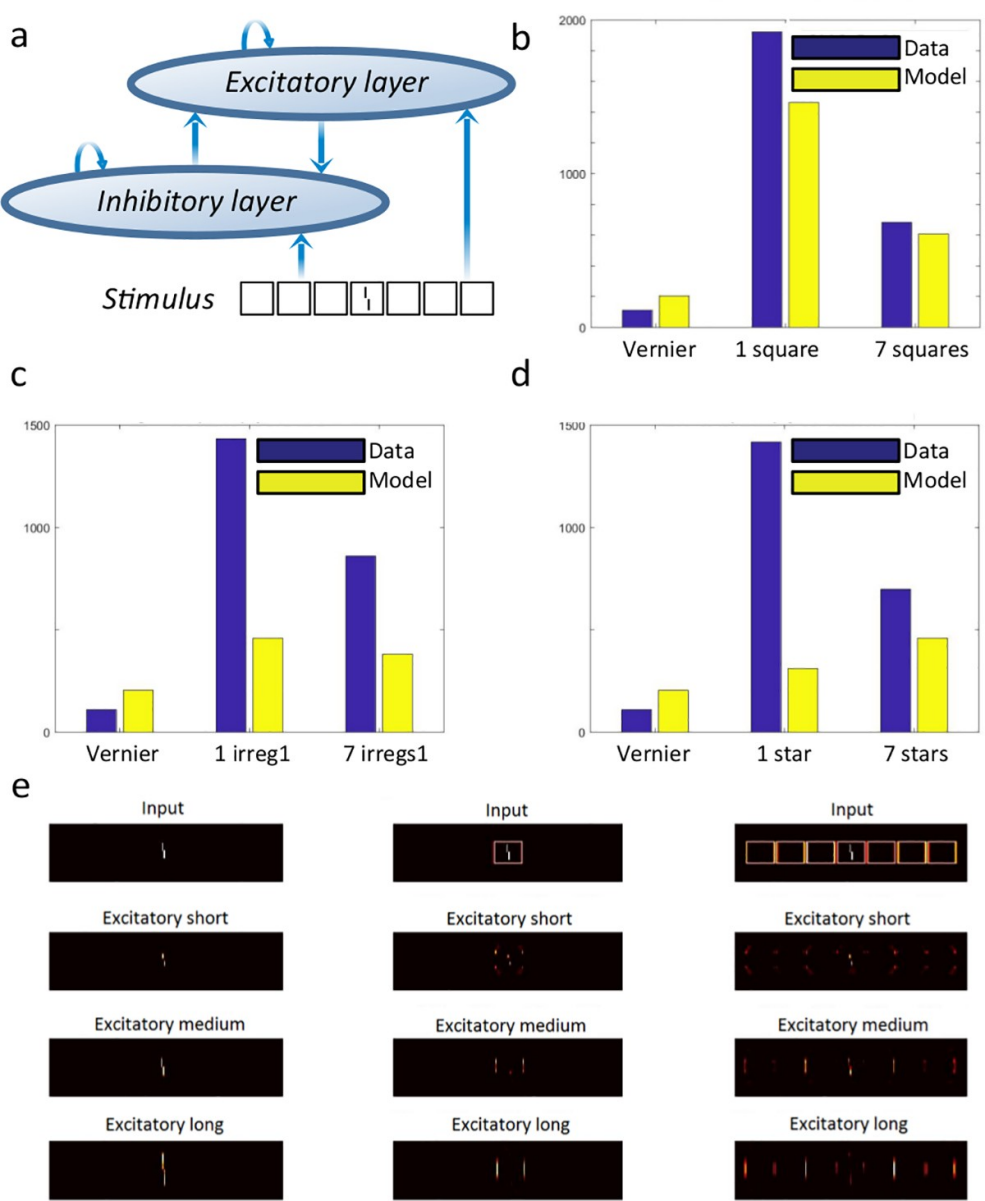
**Wilson and Cowan network with end-stopped receptive fields: a.** Structure of the network in [39] which we augmented with end-stopped receptive fields. An excitatory and an inhibitory layer of neurons are activated by the stimulus and interact with one another. The output of the excitatory layer is cross-correlated with a vernier template to measure performance. **b.** Output for the squares category (with psychometric function fitted on the squares category). In accordance with human results, performance is better in the 7 squares than in the 1 square case. **c.** Output for the irregular category (with psychometric function fitted on the squares category). Performance is marginally better in the 7 irregular1 than in the 1 irregular1 case. **d.** Output for the stars category (with psychometric function fitted on the squares category). There is no uncrowding for this stimulus. Uncrowding occurs only for specific kinds of stimuli, where element size regularities seem important. Further, performance depends strongly on which data are used for the training set (i.e., for fitting the psychometric function), suggestive of overfitting. **e.** Model output images. Columns are different stimuli: vernier, 1 square and 7 squares. The first row shows the stimuli, and the three subsequent rows show the model output for the short, medium and long end-stopped receptive fields. The crucial result is that the vernier is better represented in the short and medium populations in the 7 squares than in the 1 square conditions (i.e., uncrowding occurs). As mentioned, uncrowding occurred for very few stimuli categories. In cases that didn’t show uncrowding, the vernier representation deteriorated further when flankers were added (see results on the online repository). Note: the model outputs a cross-correlation quantifying how similar the model output is to the model output in the vernier alone condition (so the higher this cross-correlation, the better the performance). To make comparisons with human thresholds easier, we applied the same linking hypothesis as Hermens et al. [39]: we fitted a psychometric function to link model outputs to behavioural results, as explained in the main text.

We fit the psychometric function based on the model’s output for the squares category, i.e., the squares category is the training set, and used this fit to measure performance on all other stimulus categories, i.e., all other categories are the testing set. We also tried to use each of the other categories as the training set; using the squares yielded the best results. The model produces crowding: performance drops in the presence of flankers. It also produces uncrowding but only for the training set (squares) and, to a lesser extent, for the irregular1 category. Indeed, performance is better in the 7 squares than in the single square condition (Fig 8B), and marginally better in the 7 irregular1 than in the single irregular1 condition (Fig 8C). For the other categories, there is no uncrowding (see Fig 8D for an example). The choice of the training and testing sets has a strong influence on the conditions that mimic human performance. Squares and lines are the categories for which size regularity seems to play the most important role. For all other classes, there is no uncrowding, regardless of the training. This poor generalization capability suggests that the model uses idiosyncratic features of its training set rather than capturing general regularities, similar to overfitting.

#### Zhaoping’s V1 recurrent model

**Fig 9:**
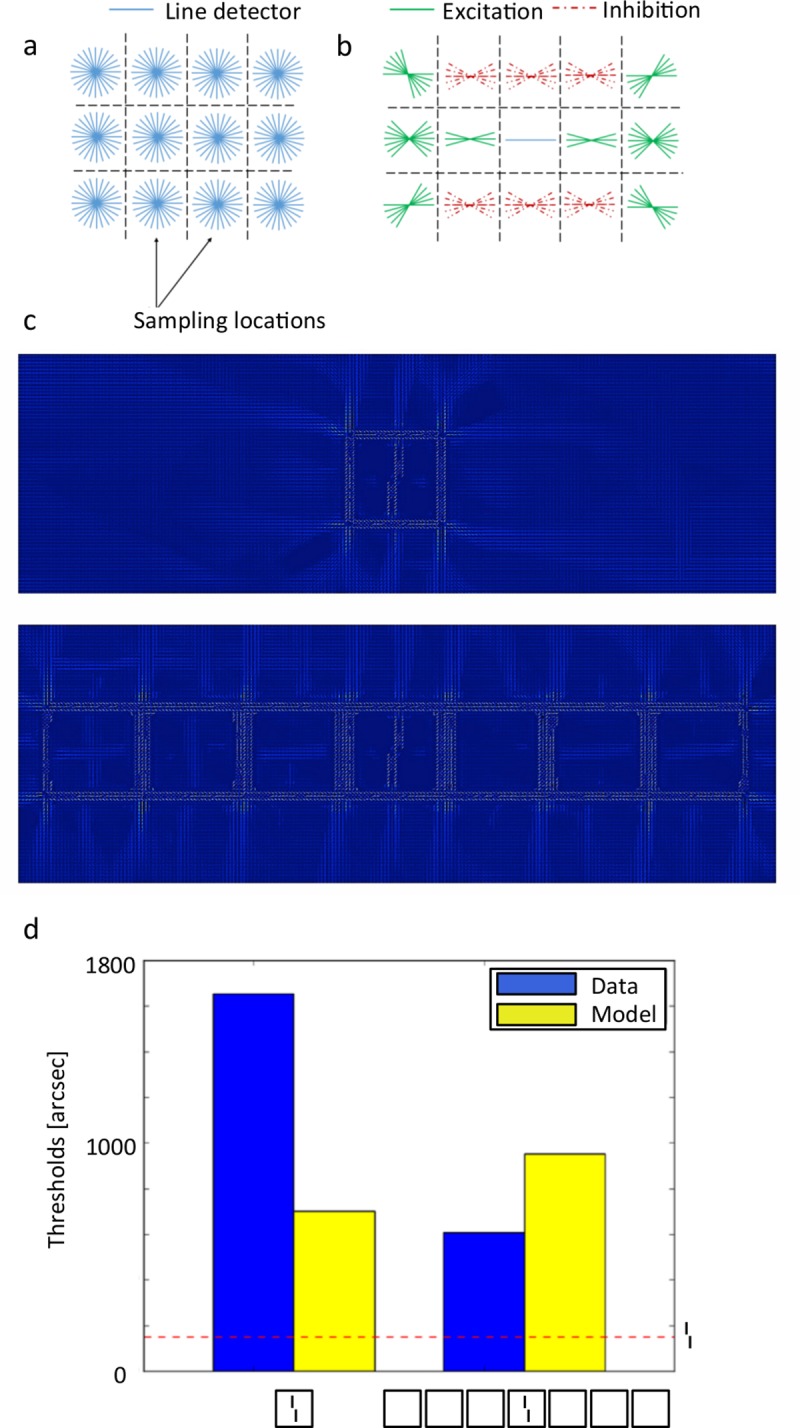
V1 Segmentation model. **a.** The input is sampled at each grid position by neurons tuned to 12 orientations, mimicking V1 simple cells. **b.** The connectivity pattern between cells depends on their relative position and orientation as shown here. Solid lines indicate excitation and dashed lines indicate inhibition. As shown, each neuron excites aligned neurons and inhibits non-aligned neurons. Each neuron has the same connectivity pattern, suitably rotated and translated. **c.** Output images for the square category. Each small oriented bar shows the maximally active orientation at this grid position. **d.** Results for the squares category. The dashed red bar shows the vernier threshold, which is matched for humans and the model. As shown, uncrowding does not occur in the model, because performance is worse for the 7 squares than the 1 square stimulus. Note: the model outputs a cross-correlation quantifying how similar the model output is to the model output in the vernier alone condition (so the higher this cross-correlation, the better the performance). To make comparison with the human threshold easier, we applied the same procedure as we did for the epitomes, i.e., we applied the following monotonic transformation to the output: “threshold-like output” = 1/”raw output”. Then we scaled the result to be in the same range as the human results. This monotonic re-scaling does not change the conclusions–the phenomenon of uncrowding cannot be altered.

This recurrent neural network model is described by Li Zhaoping [42]. The network consists of a grid of neurons tuned to 12 orientations that are linked by lateral connections that follow a specific pattern (see Fig 9A & 9B). The connectivity pattern allows the network to reproduce many experimental effects such as pop-out, figure-ground segmentation and border effects. It has also been shown to highlight certain parts of visual displays such as masked verniers [43], and we wondered if it could similarly produce uncrowding. We recoded the network from scratch following the detailed instructions and using the same parameters as in [42] and studied it as another recurrent model of early visual cortex. We ran all our stimuli and assessed performance by cross-correlating each output with the output of the vernier without flankers. The magnitude of the cross-correlation is taken as a measure of vernier offset discrimination performance. The model produces crowding but not uncrowding consistently across the dataset (see Fig 9C).

#### A variation of the LAMINART model

The LAMINART model by Cao & Grossberg [44] is a neural network capable of computing illusory contours between collinear lines. Francis, Manassi & Herzog [45] augmented it with a segmentation process in which elements linked by illusory contours are grouped together by dedicated neural populations. This dedicated neural processing operates in the same way for all conditions and plays an important role in explaining many other visual phenomena (review: [46]). This model process was intended as an implementation of a two-stage model of crowding, with a strong grouping process: stimuli are first segmented into different groups and, subsequently, elements within a group interfere. After dynamical processing, different groups are represented by distinct neural populations. Performance is determined by template matching. Importantly, crowding is low when the vernier is alone in its group (i.e., when the population representing the vernier does not also represent other elements) and high otherwise.

**Fig 10:**
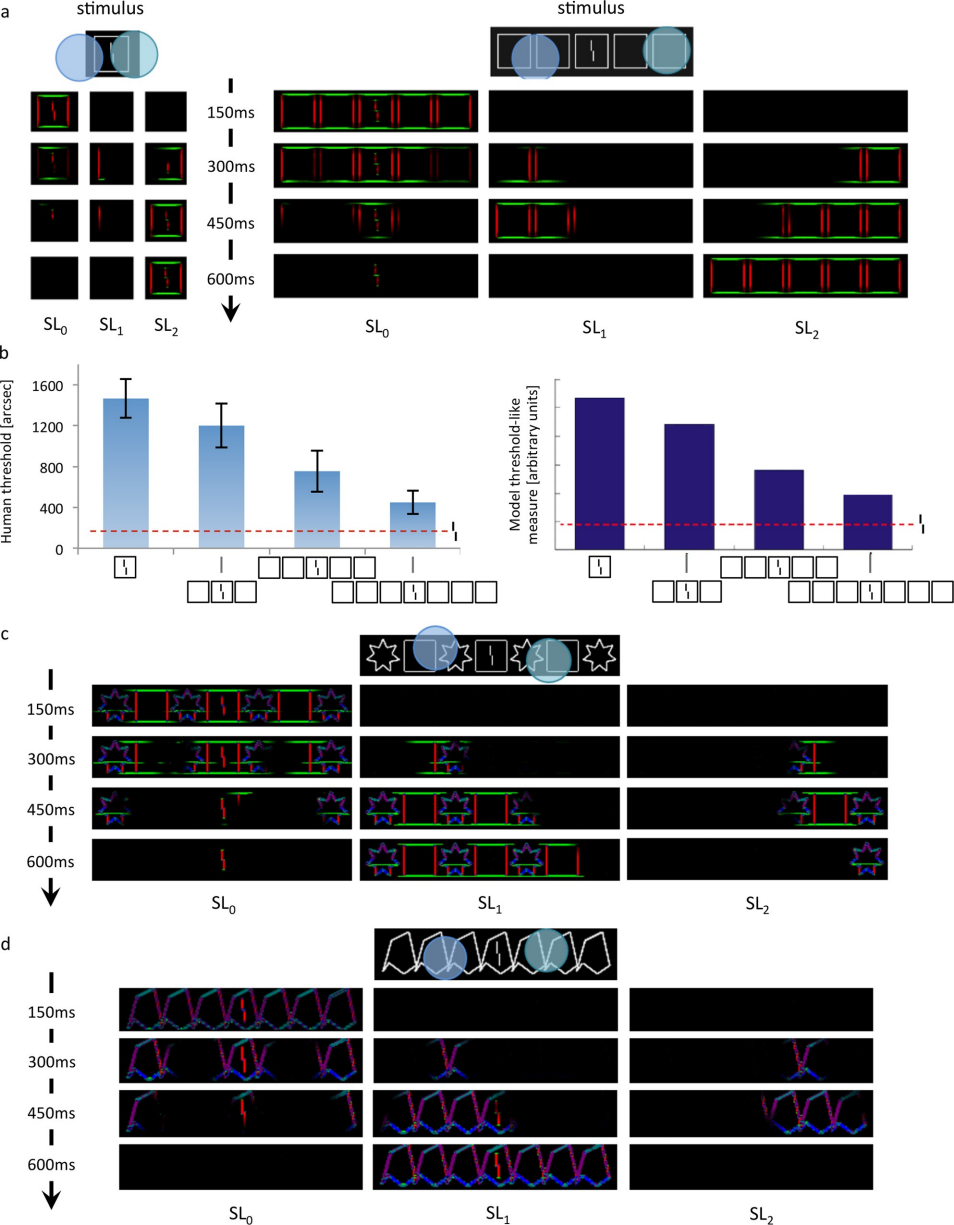
The LAMINART variation. **a**: Activity in the LAMINART model. Colors represent the most active orientation (red: vertical, green: horizontal). When a stimulus is presented, segmentation starts to propagate along connected (illusory or actual) contours from two locations marked by attentional selection signals. Visual elements linked together by illusory contours form a group. After dynamic, recurrent processing, the stimulus is represented by three distinct neural populations, one for each group. Crowding is high if other elements are grouped in the same population as the vernier, and low if the vernier is alone. On the left, the flanker is hard to segment because of its proximity to the vernier. Across the trials, the selection signals often overlap with the whole stimulus, considered as a single group. Therefore, the flanker interferes with the vernier in most trials, and crowding is high. On the right, the flankers are linked by illusory contours and form a group that spans a large surface. In this case, segmentation signals can easily hit the flankers group successfully (without hitting the vernier). The vernier thus ends up alone in its group in most trials and crowding is low. **b**: The left row shows human performance with the square flanker stimuli. The right row is the output of the LAMINART model. It fits the data very well. The same holds true for a majority of our stimuli. To compute the LAMINART’s output values, we used the same linking hypothesis as in the original description of the model [45]: template matching is used to decide if the target vernier offset is left or right, and this result is monotonically transformed into a threshold-like measure. **c**: Sometimes flankers group together (illusory contours are formed) when they should not, erroneously predicting uncrowding for this condition. **d**: Sometimes flankers group with the vernier when they should not. Here, weak illusory contours connect the central flanker and the vernier. No uncrowding can be produced for this condition because segmentation always spreads to the vernier, independently of the success of the selection signals.

The segmentation process is started by local selection signals and spreads along connected contours (Fig 10). The location of each selection signal follows a Gaussian distribution centred on a given location, with a constant standard deviation. Uncrowding occurs when the selection signals hit a group of flankers without hitting the vernier, rescuing it from the deleterious effects of the flankers. In our simulations, each stimulus is run twenty times, each time drawing a new selection signal location. The final performance is averaged over these twenty trials. Crucially, segmentation becomes easier with more flankers, because a group of many flankers connected by illusory contours produces a larger region for selection (Fig 10).

To account for the observers’ proclivity to succeed in the vernier discrimination task, the central location of a selection signal is tuned to produce the least amount of crowding for each condition. This assumption follows the idea that an observer does the best job possible in each given situation. Although this added flexibility is not present in other models, it does not constitute an unfair advantage for the LAMINART. Indeed, it is not strictly necessary in order for the model to produce uncrowding. For example, if the segmentation signals’ central location followed a uniform distribution over the whole stimulus, it would still hit a large group of flankers (without hitting the target) more easily than a small group of flankers. In summary, whenever the flankers form a wide group that can be easily segregated from the vernier, uncrowding should be produced. Hence, uncrowding is largely independent of the selection signals’ distribution.

Many stimuli in the dataset had been simulated by the model in Francis et al. [45]. Here, we improved the model by using more orientations and we ran the model on our dataset, using the template matching measure (some stimuli could not be run for reasons detailed below). Overall, the LAMINART explains the data set well (Fig 10).

More precisely, the categories circles, Gestalts, lines, octagons, squares and hexagons are all well explained. Categories irreg1, irreg2 and stars cannot be explained, but they include bars of many different orientations, and the current LAMINART simulation is only capable of handling eight orientations. We did not run the stimuli in the patternStars and patternIrregular categories because they are too large to be processed in realistic time. In general, situations where the model fails tend to be those in which the model groups elements while the data suggests it should not, leading in some cases to no uncrowding, and in other cases to excessive uncrowding. One example is when flankers (e.g., squares and stars) group together when they should not. Another example is when flankers group with the target vernier (e.g., irreg1), suggesting the need to improve the grouping mechanism itself (Fig 10).

Across all stimuli and all models, the LAMINART is by far the most successful model in this comparative study because it can explain a wide range of uncrowding results, as well as capture classic crowding effects.

#### Alexnet (Convolutional Neural Network)

**Fig 11:**
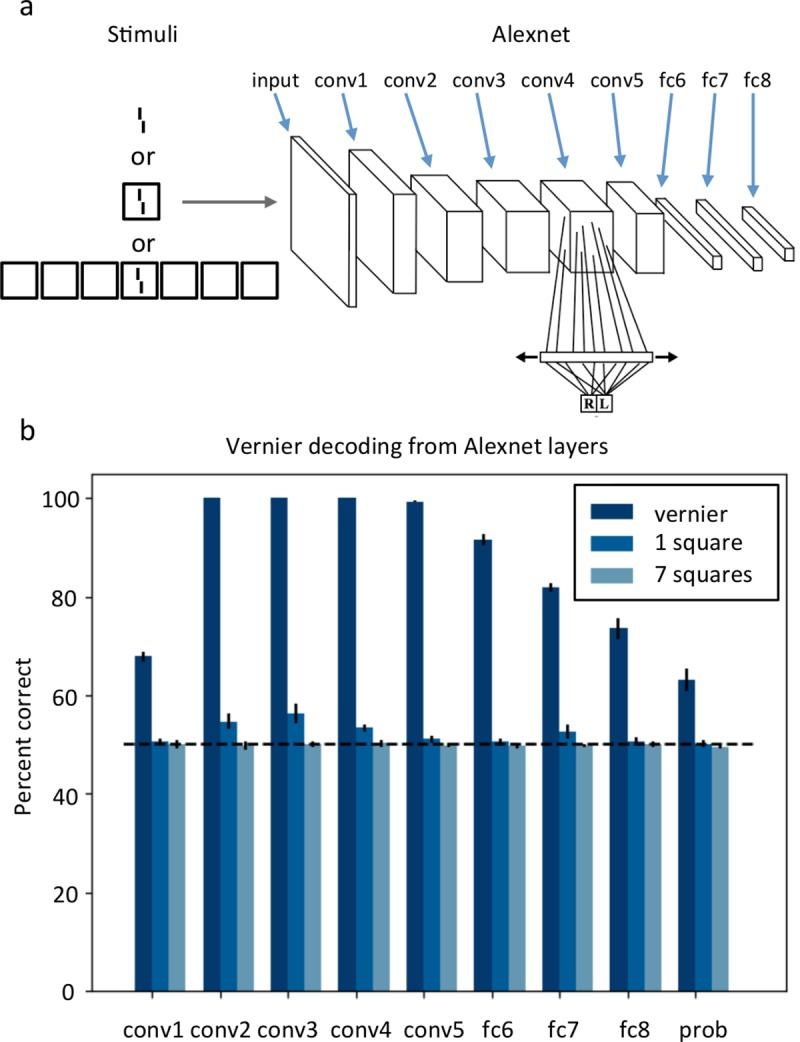
Alexnet. **a.** Stimuli consisted of either verniers, verniers surrounded by a single square or verniers with seven squares. The stimuli had varying sizes, vernier offsets and positions. Alexnet’s architecture and a classifier are shown on the right (there was a classifier at each layer). The boxes correspond to the input (leftmost box) and activated neuron layers (see [49] for the detailed architecture of Alexnet). We trained softmax classifiers on all ReLU layers following the convolution layers and the last fully connected layer to detect vernier orientation from the layer’s activity. **b.** Accuracy of softmax classifiers trained to detect vernier orientation from different layers in the deep neural network Alexnet. Across all layers, the offsets in crowded stimuli (1 square flanker) are always better detected than offsets in uncrowded stimuli (7 square flankers). This runs contrary to human performance. NB. This model only produces percent correct, there is no output image.

Deep Convolutional Neural Networks (CNNs) are local, feedforward, pooling networks. Training involves using feedback signals to adjust weights between neurons in subsequent layers. Once the network has been trained, users typically fix the weights and use the network in a feedforward manner. Given enough time and training samples, CNNs can learn any function by learning adequate weights [47,48]. CNNs fit very nicely in the standard view of vision research, in which basic features, such as edges, are combined in a hierarchical, feedforward manner to create higher-level representations of complex objects (Fig 2A). We reasoned that crowding would occur in these networks for exactly the same reason as in classic local pooling models: the target and the flankers’ representations at a given layer are pooled within the receptive fields of the subsequent layer, thus, leading to poorer performance. Although CNNs obviously compute groups such as objects or animals, these groups have no effect whatsoever on crowding of lower level features. Indeed, there are no connections from higher to lower level layers. Thus, elements far away from the vernier cannot interact with nearby elements and lead to uncrowding. To test this hypothesis, we processed the square category through Alexnet [49], a CNN trained to classify natural images with high accuracy, using Tensorflow [50]. In order to determine vernier offset discrimination in different layers, we trained classifiers to identify the vernier offset from the activations of different layers of Alexnet (Fig 11A). The classifiers had a single hidden layer with 512 units, followed by a softmax layer with two outputs, corresponding to left and right. In the training phase, we ran verniers through the network, and trained classifiers to identify the offset orientation from the different layers’ activations (which were normalized to zero mean and unit standard deviation). Each layer had its own classifier. We used all ReLU layers following the convolution layers and the last fully connected layer. A different classifier was trained for each of these layers. During the test phase, we used verniers alone, verniers flanked with a single square (crowded stimuli) and verniers with 7 squares flankers (uncrowded stimuli). Both training and testing stimuli had varying sizes, offsets and positions in the image. Fig 11 shows average performance for each layer over 6 runs. For each run, we trained a new classifier on each layer, using 250000 verniers in the training set. In the testing phase, we ran 3000 verniers, 3000 crowded stimuli and 3000 uncrowded stimuli through Alexnet. Our classifiers predicted vernier orientation from the layer activations for each of these inputs. Interestingly, our classifiers could well retrieve the test vernier orientations with 100% accuracy in almost all convolutional layers (layers 2, 3, 4 and 5). Adding square flankers deteriorated performance strongly. The single square (crowded) stimuli could be decoded only in the convolutional layers 2, 3 and 4, and in fully connected layer 7, but with much poorer accuracy than the vernier alone. Crucially, unlike in humans, the 7 squares (uncrowded) stimulus performance was always worse or equal to the performance on the single square (crowded) stimulus. Hence, the deep network produced crowding, but not uncrowding. We suggest that the mechanism leading to these results is similar to the classic local pooling account of crowding.

#### Hierarchical Sparse Selection (HSS)

**Fig 12:**
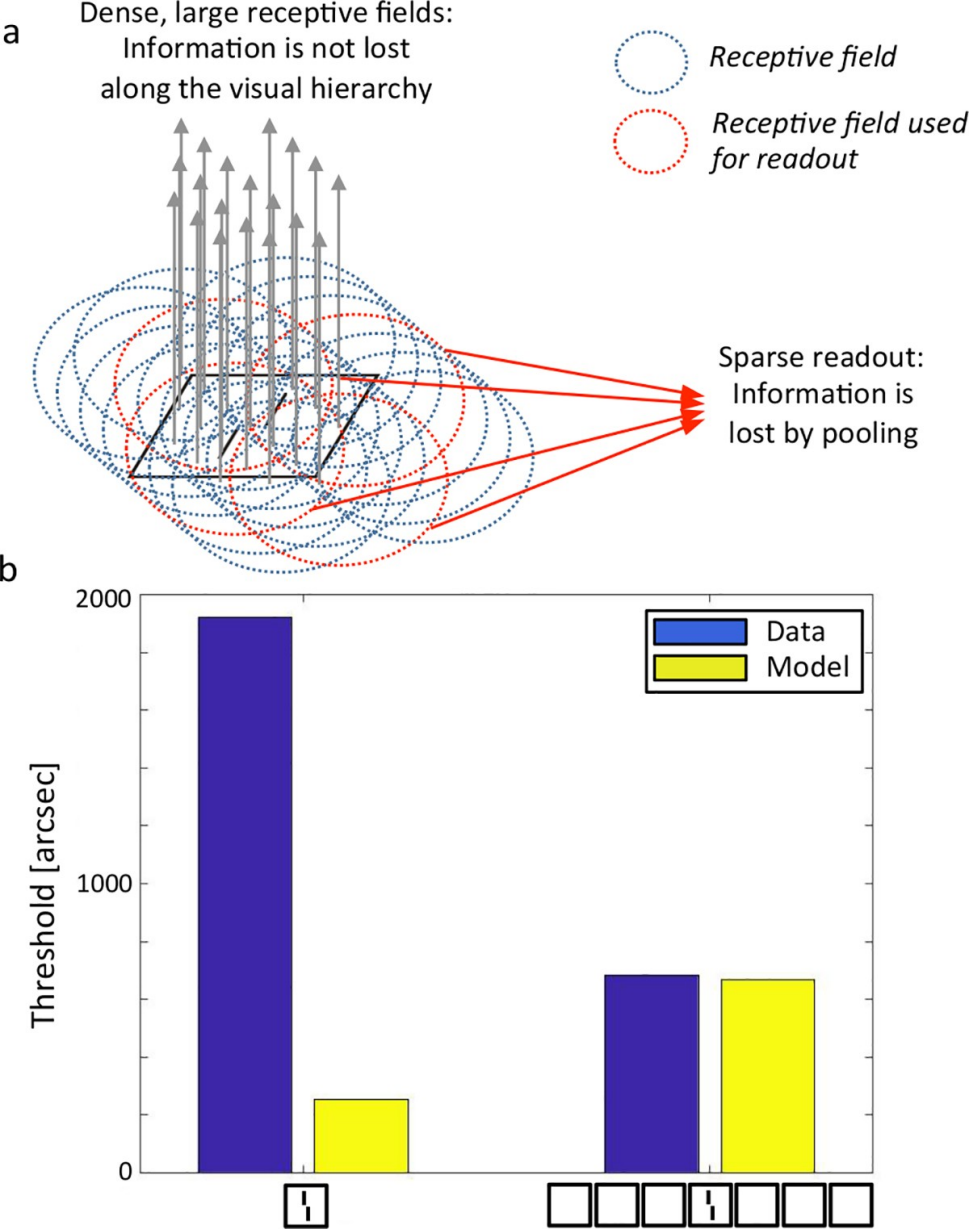
Hierarchical Sparse Selection model. **a.** The model posits that receptive fields along the visual hierarchy are large and dense. This allows for “lossless” transmission of information through the visual system. For instance, the offset of the vernier in this illustration is not corrupted by pooling thanks to the density of the receptive fields (blue and red circles). Crowding occurs because, when we try to access information, only a few sparse receptive fields are used for readout (red circles). Hence, crowding occurs at readout because of sparse sampling of receptive fields. This sparse readout can occur at any stage of visual processing, from low-level features (shown here) to faces. **b.** Uncrowding does not occur in the Hierarchical Sparse Selection model because performance is worse for the model on the 7 squares than the 1 square condition, contrary to human performance. NB. This model only produces a scalar output, there is no output image.

This model was described by Chaney, Fischer & Whitney [51]. In a series of experiments, it was shown that in spite of difficulty identifying a crowded target, crowding does preserve some information about the target, i.e., information is rendered inaccessible but not destroyed (see [8,9] for reviews). For example, a face surrounded by other faces cannot be explicitly identified, but information about its features can nevertheless survive crowding and contribute to the perceived average of a set of faces [52]. To accommodate these results, Chaney et al. proposed that information is not lost along the visual processing hierarchy. Instead, crowding occurs because readout is sparse. Specifically, given a feature map representing a stimulus, only a subset of the neurons from this map can be used to decode the target, which leads to crowding’s deleterious effects (Fig 12A).

Using the author’s code, we tested all our stimuli and found that crowding could be explained, but uncrowding did not occur in the model (Fig 12B). Originally, the model was used to detect crosses, triangles and circles. We modified the model’s readout layer to classify vernier orientation, which was achieved with 99.13% accuracy (the rest of the model does not need any change to accommodate new stimuli). Then, we dropped 75% of the neurons for the sparse readout, which led to a vernier classification accuracy of 81.48%. We tested all our stimuli by asking the model to classify the vernier orientation, first without dropping any neurons, then with 75% of the neurons dropped for the sparse readout, as we did for the verniers. For all stimuli, performance dropped with the sparse readout. For example, the 1 square condition was classified with 93.35% accuracy when all neurons were used, and this dropped to 75.55% with sparse readout. The 7 squares condition had a similar profile, but classification accuracy was worse than for the 1 square condition (71.73% with all neurons and 59.23% with sparse readout). This pattern of results was found in all stimulus categories: sparse readout impaired performance, and adding more flankers impaired performance too. Thus, there was crowding but no uncrowding. We would like to mention that Chaney et al. argue that uncrowding can in fact be explained, if the target and flanker are represented in different feature maps, which are however not implemented at the moment. In essence, visual stimuli are segmented into different feature maps (this must happen early in the visual pathway to explain the low-level vernier results), and subsequently the HSS model applies within feature maps, on this pre-segmented input.

### Models tested elsewhere

The following models were not implemented here, but we mention them for completeness.

#### Saccade-confounded summary statistics

Nandy & Tjan [53] proposed a model linking summary statistics to saccadic eye movements: crowding is proposed to occur because the acquisition of summary statistics in the periphery is confounded by eye-movement artifacts. This leads to inappropriate contextual interactions in the periphery and in this way produces crowding. For the present purposes this is not directly relevant, because foveal and peripheral uncrowding results are qualitatively identical [11], which the saccade-confounded summary statistics model cannot explain since it suggests that crowding can only occur in peripheral regions. Furthermore, it is not clear how uncrowding can occur in this model.

#### Population coding

This kind of model was first described by Van den Berg, Roerdink, & Cornelissen [54]. A similar model was proposed by Harrison & Bex [55]. Both models elegantly produce both pooling and substitution behaviour by assuming that an element’s orientation is represented by a population code: a probability distribution of its orientation. When many elements are present, the population codes interfere and disturb the target element’s representation, which leads to crowding. This interference depends on distance and is usually modeled as a 2D Gaussian. Dayan & Solomon [56] also proposed a model in which elements are represented as probability distributions. They added a Bayesian process to account for the accumulation of evidence over time. Their model captures local crowding effects similarly to Van den Berg et al. and Harrison & Bex’s models: the interference comes from the representations of neighbouring elements deleteriously affecting each other. This model and the one by Van den Berg and colleagues cannot handle images as input and thus could not be tested with our stimuli.

We have shown elsewhere that the Harrison & Bex [55] implementation cannot explain uncrowding [57]. Agaoglu & Chung [58] showed that the interaction between elements depends on which of them is considered as the target for report. Hence, the crowding interference between elements in the display depends on the task, which is not easily incorporated in the models without a dedicated process. Van den Berg et al. [54] suggested that elements do not interfere when they are represented in different perceptual groups, similar to the LAMINART model. Similarly, Harisson & Bex [55] have suggested that a preprocessing stage determining which elements interfere is needed.

#### Fourier model

**Fig 13:**
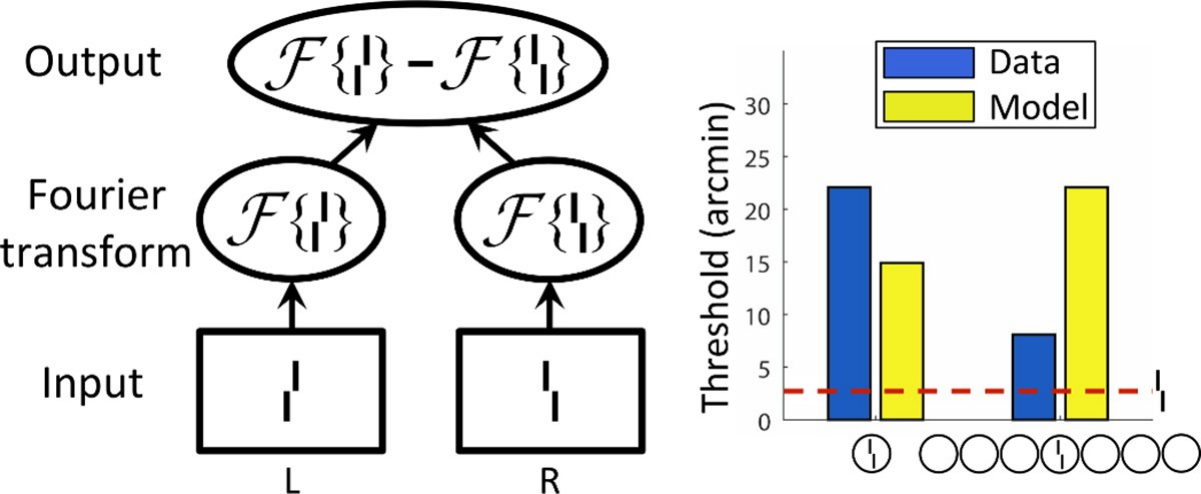
Fourier model. **Left.** The Fourier model computes Fourier transforms for the left- and right-offset versions of each stimulus. If these transforms are very different, crowding is low because the offset direction is easy to decode in Fourier space [15]. **Right.** Output of the Fourier model. The model failed on most stimuli [15]. NB. This model only produces a scalar output, there is no output image.

The Fourier transform is sensitive to global aspects of spatial configurations because it is based on periodic features. Even if it was never explicitly proposed to explain crowding, it may capture some effects of uncrowding that have to do with regularities in the stimulus. Previously [15,41], we used a Fourier-based model and tested it on the entire dataset. Essentially, this is a texture-like model, assuming that the brain Fourier transforms the visual input. Repetitive structures, such as arrays of squares are more compactly coded in the Fourier space than the 2D space. We restate the results here for comparison with the other models. The model first bandpass-filters the stimuli (passing a small range of frequencies at all orientations), then computes the Fourier transforms of the filtered left- and right-offset cases for each stimulus. Similarly to what was done to measure performance of Zhaoping’s recurrent V1 model, these are cross-correlated with the filtered versions of the verniers without any flankers and the magnitude of the cross-correlation is taken as a measure of vernier offset discrimination performance. This process is repeated over all possible pass-bands (which is finite given a fixed image size) until the pass-band yielding performance most similar to humans is found. Across the dataset, this approach failed to reproduce the data (see Fig 13), suggesting that such a simple use of global regularities in the display is insufficient to explain crowding. Depending on the set of Gabor filters, uncrowding occurred for certain stimuli, but this was never consistent over several stimulus types, which is suggestive of overfitting. With one set of filters the lines category could be explained, with another the Gestalts category could be explained.

## Discussion

For decades, crowding was thought to be fully determined by nearby elements. For this reason, target elements were presented only with a few nearby elements, and models were local in nature. However, experiments of the last two decades have shown that elements far beyond Bouma’s window can strongly affect performance. Crowding can become stronger [12] or weaker [10,13–16] when elements are presented outside Bouma’s window. Hence, local models cannot provide a complete account of crowding. In addition to spatial extent, it is the specific stimulus configuration that determines crowding. Configurational effects are not small modulations of crowding but have large effect sizes and, more importantly, can qualitatively change the pattern of results. For example in Fig 2B, performance changes in a non-linear U-shaped fashion with best performance for the unflanked target, strong crowding for few flankers, and weaker crowding when flankers make up a regular configuration.

A major question is at which computational level crowding occurs. In local models, only nearby elements interfere with target processing, often due to low level mechanisms such as pooling. In global models, features across the entire visual field are potentially important. Global interactions may be restricted to low level features, such as the orientations of the stimulus elements. At the other extreme, explicitly computing objects (such as the squares in Fig 2) may turn out to be necessary. Likewise, face crowding may or may not necessitate the explicit computation of faces [8,52,59,60]. For this reason, some global models explicitly compute grouping-like aspects. Only elements within a group interfere with each other. Classically, models restricting themselves to lower level features are given priority because they offer more parsimonious explanations.

### Model comparison

Here, we investigated all available models suited to explain the global aspects of crowding.

**Fig 14:**
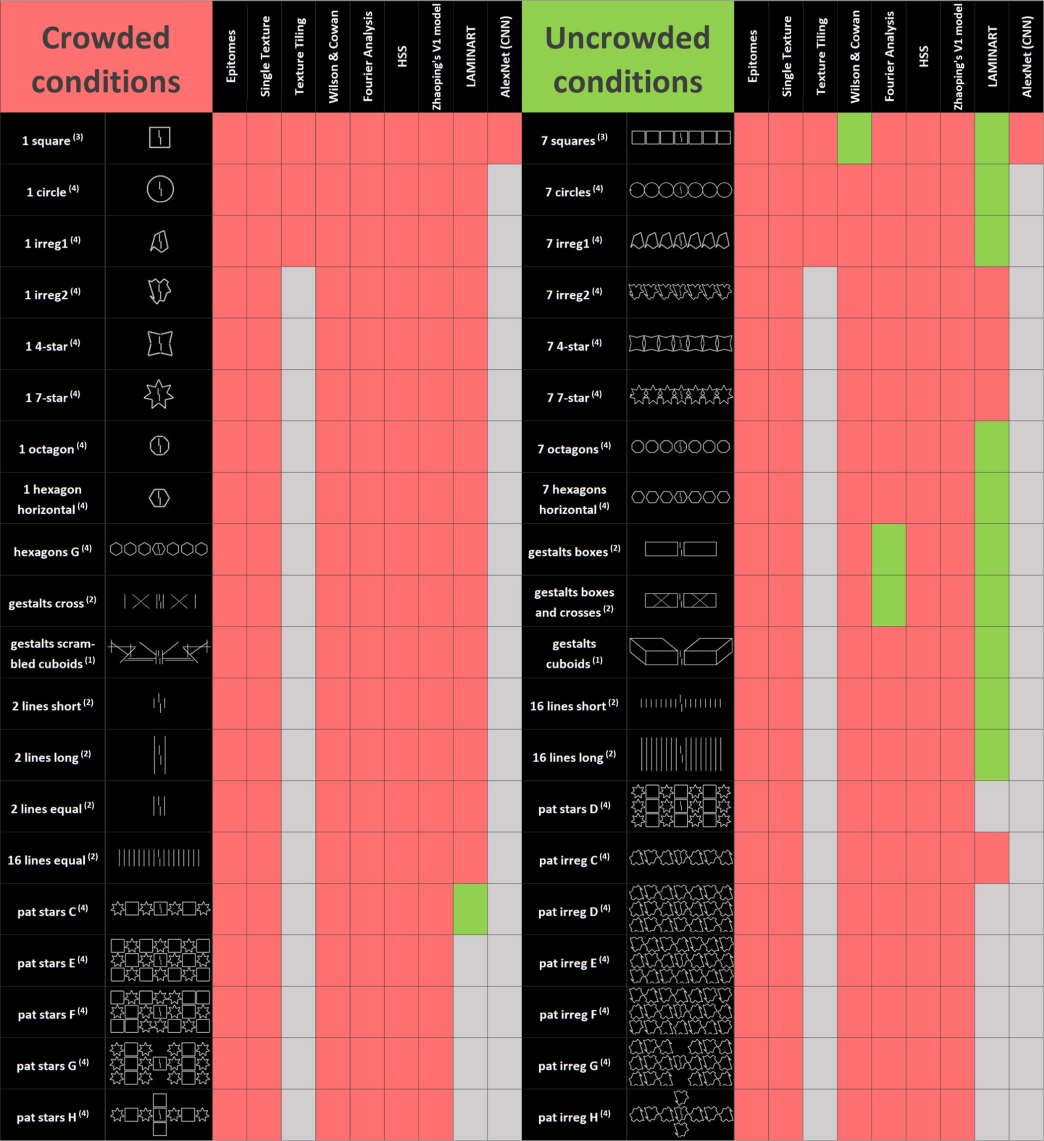
Summary of results. Results for all models (columns). In black, the left panel displays all crowding stimuli and the right panel displays all uncrowding stimuli (i.e., better performance when extra elements are added to the crowded condition) as observed in human data (rows). Superscript numbers indicate which publication the results are taken from (1: Sayim, Westheimer & Herzog [17]; 2: Manassi et al. [11]; 3: Manassi, Sayim & Herzog [19]; 4: Manassi et al. [15]). Red indicates that the model predicts crowding, green indicates uncrowding and gray indicates that we did not run the model on the stimulus. A perfect model would have only red in the left half of the table and only green in the right half. Only the LAMINART is capable of producing uncrowding consistently. Fourier and the Wilson-Cowan network produce uncrowding, but suffer from overfitting (see discussion). For these two models, we provide the results for the best parameters. For example, the Wilson and Cowan with different parameters can explain the lines category but then it cannot explain the squares and irregular1 categories.

**Fig 15:**
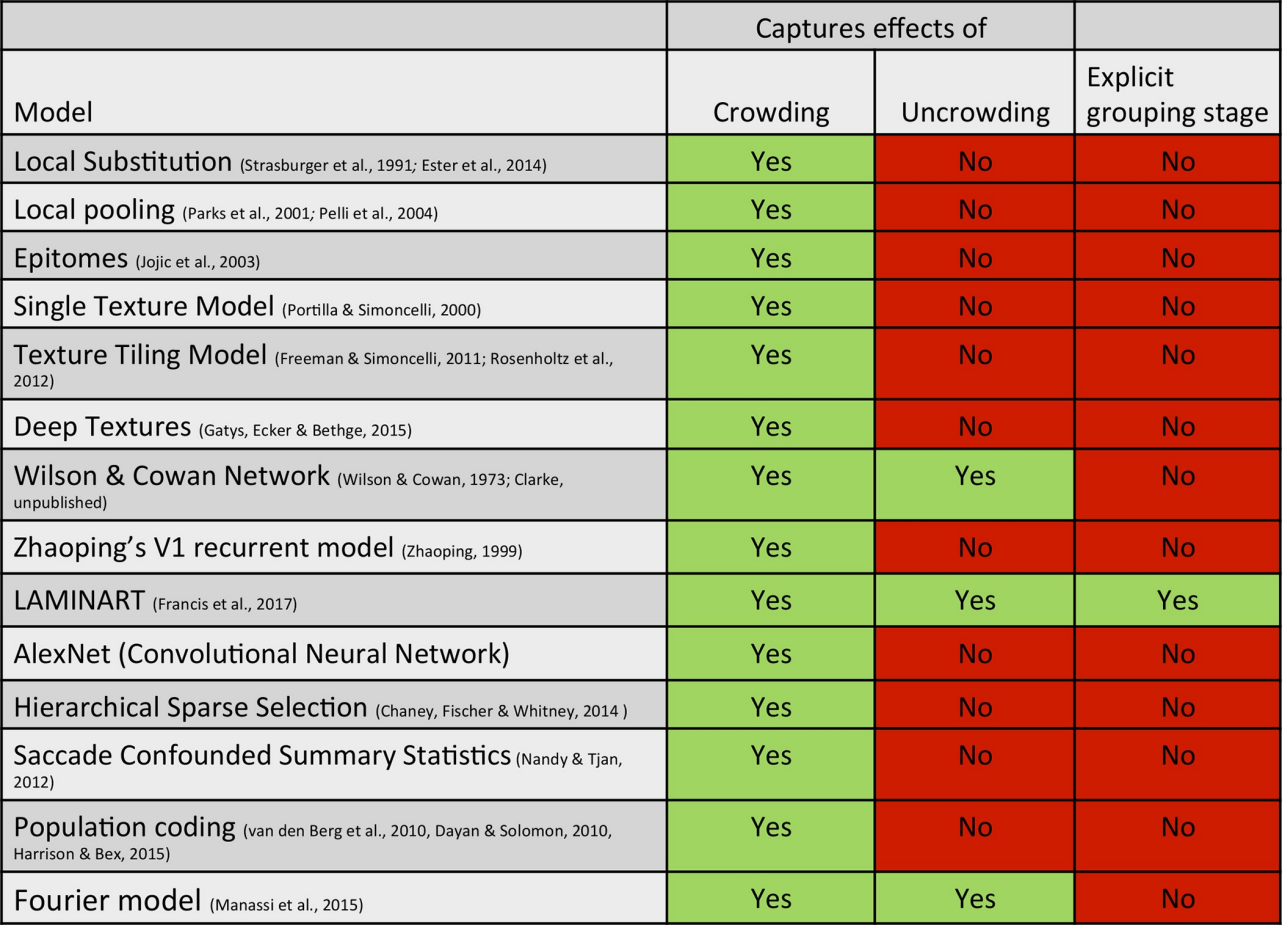
Model comparison. All models produce crowding, but only the Fourier, Wilson and Cowan and LAMINART models produce uncrowding. The Fourier and the Wilson and Cowan model overfit and thus do not capture general principles. The LAMINART is the only model that explicitly computes grouping like aspects and segments the image into different layers.

All models (leaving aside Deep Textures, which was never proposed to explain crowding with laboratory stimuli) produced crowding comparable to the human data. However, only the LAMINART model was consistently able to produce *un*crowding (Figs 14 & 15). The Wilson and Cowan network produced uncrowding only for the squares category (and to a lesser extent for the irregular1 category). The Fourier model produced uncrowding only for the Gestalts and lines stimuli. In both models, uncrowding depended heavily on parameter values, a signature of overfitting. In the Wilson and Cowan network, the end-stopped receptive fields led to grouping elements of similar size, but this did not generalize to explain other global effects.

We think there are principled reasons why most models cannot reproduce most of the global uncrowding findings. First, the effects of global configuration (Fig 2C) operate on a much higher level than most models can capture. To phrase it this way, we think that human performance is based on global configurations and not on simple hidden sub-regularities, such as repeating patterns or simple summary statistics. Second, as Wallis et al. [37] put it: “Based on our experiments we speculate that the concept of summary statistics cannot fully account for peripheral scene appearance. Pooling in fixed regions will either discard (long-range) structure that should be preserved or preserve (local) structure that could be discarded. Rather, we believe that the size of pooling regions needs to depend on image content”. For this reason, we think that performance in crowding cannot be explained simply as a by-product of basic spatial processing, e.g., by summary statistics. In contrast, which elements interfere seems to depend on the global stimulus layout. We propose that the LAMINART can consistently produce uncrowding because it can deal with this requirement by incorporating a grouping-like process: elements linked by illusory contours are grouped together and segmented from elements in other groups. Interference happens only between elements within a group.

Another way to approach the importance of grouping for crowding is that it provides extra information that makes one condition inherently easier than another. Vernier acuity tasks are often thought to be mediated by the responses of one or more feature detectors. Each feature detector might itself look like a vernier offset, or might be similar to an orientation detector such as a Gabor. Regardless, correct performance at the vernier task requires precise placement of the detector; a slightly misplaced detector can easily give the wrong answer, particularly when the vernier is flanked by other stimuli. Crowding induces location uncertainty. Any information that can help correctly place the detector–essentially any cue to the right position–would improve performance. Strong stimulus grouping could be one such cue (Rosenholtz et al., under review). In this case too, it is crucial to understand how the brain groups visual elements across the entire visual field.

The LAMINART model links elements by illusory contours, which is a rather basic grouping mechanism. It remains an open question whether more complex features are necessary to explain crowding/uncrowding such as an explicit computation of objects, e.g. squares, faces etc. For example, can the irregular shapes category be explained with simple contour integration? Likewise, it remains an open question whether face crowding can be explained without the explicit computation of faces.

In the LAMINART model, the grouping and interference processes are separate. Alternatively, grouping and interference may be intimately linked. One possibility is that the groups correspond to optimal statistical representations. For example, elements may form a group when they can be well compressed by summary statistics. In this scenario, grouping is part of the summary statistics process itself. There are probably many other ways in which grouping may play a role.

A major problem with the grouping approach is the lack of a well-defined, objective measure of grouping. If there is no objective measure, groups can be chosen ad hoc to explain experimental results, leading to circular explanations. As a first step towards an objective measure of grouping, subjective measures (i.e., asking observers to report what they feel belongs to a group) can complement studies. Such subjective ratings about perceptual groups have correlated well with psychophysical performance levels [11].

### Future models

As we have shown, none of the current models can fully explain (un)crowding. What would the model of the future look like? What components are crucial?

First, as mentioned earlier, we can rule out local models because elements across large parts of the visual field influence perception of the target.

Second, to explain the complex effects of spatial configurations in crowding, our results suggest that grouping-like, mid or higher level aspects need to be incorporated in a model. However, the exact nature of this process is unknown. For example, it may or may not be that mid-level processing is sufficient. In addition, the incorporation of higher level processes does not exclude the additional use of summary statistics and other lower level components. The grouping stage is difficult to study because of the seemingly infinite number of possible visual configurations. We believe that new tools are needed to help navigate the huge search space effectively. For example, Van der Burg, Olivers, & Cass [61] have proposed a genetic algorithm to find configurational features important for crowding.

Third, we cannot rule out feedforward models. Indeed, it is a mathematical fact that any recurrent model can be “unfolded” into a feed-forward network [62–64]. However, these feedforward models are usually extremely large and computationally expensive. For this reason, we suggest that models with feedback connections are much more likely to be able to explain how complex spatial configurations influence target processing. For example, higher level grouping processing, such as computing the squares and grouping them together, may feed back to lower level processing of the target, i.e., the vernier. Support for this hypothesis comes from the finding that the Alexnet CNN could not produce uncrowding, presumably because high-level features cannot influence low-level processing.

Fourth, the nature of interference remains unclear. One option is that interference occurs during complex spatial processing by an unknown mechanism. Another option is that the classic interference mechanisms operate after complex spatial processing is accomplished. For example, pooling may occur only for grouped elements. In the same line of reasoning, Chaney et al. [51], Van den Berg et al. [54] and Harrison & Bex [65] noted that adding a grouping stage to their interference mechanism may help explain a wider range of results. Combining complex spatial processing with good interference mechanisms may, therefore, allow for a happy marriage between interference- and grouping-based mechanisms leading to a truly unified model of crowding.

### Conclusion

The global stimulus configuration plays a crucial role in crowding, which cannot be captured by local models. For this reason, we propose that models of crowding need to include grouping like processes. While our results show that none of the current models lacking a grouping process can explain the global uncrowding phenomena, they may be good candidates for a potential second, interference stage.

How are basic features of the visual field grouped to form objects? The most successful model we analyzed, the LAMINART variation, suggests that this is done by linking features together by illusory contours. Further work is needed to assess how far this mechanism can go and what alternative or additional components are necessary, such as summary statistics. For example, the groups may correspond to optimal statistical representations (elements that can easily be compressed using summary statistics would form a group).

Most importantly, large scale, configurational effects are not restricted to visual crowding with vernier targets. Uncrowding occurs also for letters and Gabors [66], as well as in audition [23] and haptics [24]. Similar effects are found in backward masking [67] and overlay masking [17,68]. Hence, crowding is a special case of contextual processing. Vision research has largely missed these aspects because of the use of well-controlled stimuli, which are usually presented in isolation or with only a few nearby flankers. Our results suggest that in order to understand vision in general, a mid-level, contextual processing stage is inevitable.
